# Cytological Anal Squamous Intraepithelial Lesions Associated with Anal High-Risk Human Papillomavirus Infections among Men Who Have Sex with Men in Northern Thailand

**DOI:** 10.1371/journal.pone.0156280

**Published:** 2016-05-26

**Authors:** Darin Ruanpeng, Suwat Chariyalertsak, Quanhathai Kaewpoowat, Taweewat Supindham, Jongkolnee Settakorn, Kornkanok Sukpan, Utaiwan Utaipat, Toshiyuki Miura, Natthapol Kosashunhanan, Pongpun Saokhieo, Radchanok Songsupa, Antika Wongthanee

**Affiliations:** 1 Research Institute for Health Sciences, Chiang Mai University, Chiang Mai, Thailand; 2 Division of Infectious Diseases, Department of Medicine, Faculty of Medicine, Chiang Mai University, Chiang Mai, Thailand; 3 Department of Pathology, Faculty of Medicine, Chiang Mai University, Chiang Mai, Thailand; 4 Department of Clinical Medicine, Institute of Tropical Medicine, Nagasaki University, Nagasaki, Japan; The University of New South Wales, AUSTRALIA

## Abstract

**Background:**

Anal cancer, one of human papillomavirus (HPV) related malignancies, has increased in recent decades, particularly among men who have sex with men (MSM) and HIV-infected (HIV+) persons. We aimed to explore the prevalence of anal squamous intraepithelial lesions (ASIL) using Papanicolau (Pap) screening among MSM in northern Thailand and its associated factors.

**Methods:**

Two hundreds MSM aged ≥18 years reporting receptive anal intercourse in the prior 6 months were recruited from July 2012 through January 2013. Medical history and behavioral data were collected by staff interview and computer-assisted self interview. Anal Pap smear, HPV genotyping, and HIV testing were performed. Two pathologists blinded to HPV and HIV status reported cytologic results by Bethesda classification.

**Results:**

Mean age was 27.2 years (range 18–54). Overall, 86 (43.0%) had ASIL: 28 (14.2%) with *atypical cells of undetermined significance* (ASCUS), 1 (0.5%) with *atypical squamous cells—cannot exclude high-grade squamous intraepithelial lesion* (ASC-H), 56 (28.4%) with *low-grade squamous intraepithelial lesion* (LSIL), and 1 (0.5%) with *high-grade squamous intraepithelial lesion* (HSIL). ASIL was associated by univariate analysis (*p* ≤0.05) with older age, gender identity other than bisexual (i.e., gay men and transgender women), rectal douching, anal symptoms, genital warts, HIV positivity, and high-risk-HPV infection. However, on multiple logistic regression ASIL was associated only with high-risk HPV type (*p* = 0.002) and HIV infection (*p* = 0.01).

**Conclusions:**

ASIL is quite common in high-risk MSM in northern Thailand and is associated with high-risk HPV types and HIV infection. Routine anal Pap screening should be considered, given the high frequency of ASIL, particularly in the HIV+. High resolution anoscopy (HRA), not done here, should be to confirm PAP smears whose sensitivity and specificity are quite variable. Timely HPV vaccination should be considered for this population.

## Introduction

An increasing incidence of squamous cell cancer of the anus has been observed in diverse countries worldwide in the past couple decades [1] [2] [3]. Populations at risk include men who have sex with men (MSM) and human immunodeficiency virus-infected (HIV+) persons. The major risk factor for anal cancer in MSM is receptive anal intercourse [4] [5], which is also associated with infection by the human papilloma virus (HPV) [5] [6] [7]. While the estimated annual incidence of anal cancer in the general population is 2 per 100,000 [8], meta-analysis has estimated its incidence to be 5 per 100,000 among HIV-negative (HIV–) MSM and 46 per 100,000 in the HIV+ MSM [9].

A number of strains of HPV (sometimes referred to as *serotypes* or *genotypes* when determined by serologic assay or genetic sequencing, respectively, but simply as *types* in this report) are considered high-risk for their association with various forms of cancer, for which the strength of that association for anal cancer is second only to that for the cervix in women [10]. The pathogenesis of both anal and cervical cancers are believed similar: starting with HPV infection, which after persistence induces low-grade lesions, which progress to high grade-intraepithelial lesions if spontaneous clearance does not occur, and eventually in some becoming invasive neoplasms [10] [11]. However, unlike screening for cervical cancers, routine screening for anal cancer remains controversial [12].

We investigated the prevalence of abnormal anal cytology in MSM in northern Thailand in order to identify associated clinical, behavioral, and viral factors that would be useful in developing policies for prevention, screening, and management of anal cancer in this population.

## Materials and Methods

Study data and specimens were collected at the PIMAN Center, a HIV voluntary counseling and testing unit for MSM, including bisexual men (BM), gay men (GM), and transgender women (TGW), located in Chiang Mai city of northern Thailand.

### Ethics

Prior to study initiation, the Ethics Committee of the Research Institute for Health Sciences, Chiang Mai University, reviewed and approved its protocols, questionnaires, and written informed-consent forms for informing and soliciting PIMAN Center clients voluntarily to enter the study. Study participants were informed of their results for study testing for HIV and HPV, followed by appropriate counseling and treatment, if indicated. Testing for other sexually transmitted diseases (STD) at the PIMAN Center are an additional service provided only upon client request, and when diagnosed or suspected, clients are treated at the PIMAN Center and/or referred elsewhere for treatment or further diagnosis. Participants with abnormal anal cytological findings were advised to seek medical care for appropriate follow up. This publication accords with community standards and was approved by the Ethics Committee.

### Study recruitment and data collection

A companion paper from this same study and volunteer cohort reported the demographic, sexual, behavioral, medical history, virology, and coverage of HPV types by various HPV vaccines [13], and should be consulted for more details of methods and findings than those reported here. In brief, approximately 400 MSM clients attending the PIMAN Center from July 2012 through January 2013 were invited to participate in the study, whose inclusion criteria included self-identification as a BM, GM, or TGW, age ≥18 years, and having practiced receptive anal intercourse in the prior 6 months. A total of 200 MSM consented to enroll. Participant information was gathered by initial screening questions, computer-assisted self-interview (CASI), and private interview by study researchers. CASI data was not analyzed until the enrollment was completed. When CASI and enrollment interview produced discordant reports on sexual practices in the prior 6 months, enrollment interview was used to satisfy the corresponding inclusion criteria and retention in the analysis.

Gender identity as BM, GM and TGW was determined by self-report on CASI. The exposures and factors of interest for association with anal squamous intraepithelial lesions (ASIL) were demographic (age, occupation, gender identity), sexual behavioral (gender identity, sexual role, sexual-debut age, number of partners in past 6 months, condom use, rectal douches), and clinical (genital warts, probable symptoms of STDs, estrogen use, smoking, and HIV status), as previously noted for possible relationship with HPV infection and anal cancer [4] [5] [10]. Participants considered to have a probable STD sign or symptom were those reporting any of the following: anal sore(s) or itch, pain or bleeding with defecation, tenesmus, mass in the anal area, burning upon urination, penile discharge, or testicular pain. Abnormal anal symptom(s) was (were) defined as any reported anal sore or itch, pain or bleeding upon defecation, tenesmus, or mass in the anal area. Abnormal penile symptom(s) was (were) any burning upon urination or penile discharge. Unless specified as insertive or receptive, anal intercourse was defined as sexual activity through the anal canal by either role. Condoms were considered used when reported by the participant as worn by either partner regardless of insertive or receptive role.

### Specimen collection

Anal specimens for cytologic (Pap smear) analysis and for HPV genotyping were collected by study physicians, who introduced a saline-moistened nylon swab into the anal canal up to 5 cm and circularly rotated it for one minute, applying gentle pressure upon the canal walls. The swabs were then inserted promptly into for ThinPrep liquid based cytology preparation (Hologic, Boxborough, MA, USA). Swab specimens were assigned unique identification (ID) codes which differed from both study ID numbers and from routine ID numbers of PIMAN Center clients.

### Cytologic analysis

Cytologic diagnoses were assigned independently by two experienced pathologists from the Department of Pathology, Chiang Mai University, who were blinded to data from the CASI, face-to-face interviews, HPV, and HIV testing results. The final consensus of both pathologists for a specimen’s anal cytology were classified using the 2001 Bethesda system for cervical cytology as 1) *negative for intraepithelial lesion or malignancy*, 2) *atypical squamous cells of undetermined significance* (ASC-US), 3) *atypical squamous cells—cannot exclude high-grade squamous intraepithelial lesion* (ASC-H), 4) *low-grade squamous intraepithelial lesion* (LSIL), 5) *high-grade squamous intraepithelial lesion* (HSIL), or 6) *squamous cell carcinoma* [14]. The College of American Pathologists and the American Society for Colposcopy and Cervical Pathology recommend classifying anal epithelial abnormalities similarly to that used for cervical (Pap smear) cytology in the Bethesda system [15]

### HPV detection and genotyping

Details of study methods for DNA extraction, polymerase-chain-reaction (PCR) amplification, and typing of HPV from anal swab specimens are available elsewhere [13]. The thirteen HPV types 16, 18, 31, 33, 35, 39, 45, 51, 52, 56, 58, 59, and 68 were defined herein as high risk HPV (“hr-HPV”), as there is evidence of human carcinogenicity for the first twelve listed (sometimes referred to as Group 1), or probable human carcinogenicity for the final type 68, sometimes referred to as Group 2a [10] [16] [17]. The other twenty-four (6, 11, 40, 42, 53, 54, 55, 61, 62, 64, 66, 67, 70, 71, 72, 73, 81, 82, 83, 84, IS39, and CP6108) included those sometimes classified as Group 2b for possible carcinogenicity, and Group 3 as not classifiable as to their carcinogenicity for humans (and not listed in a monograph of the International Agency for Research on Cancer [10]). These 24 were classified herein as “non-hr-HPV”. Participants with any one or more of the hr-HPV types were classified herein as hr-HPV infected. Those with only non-hr-HPV types were classified as non-hr-HPV infected.

### HIV testing

Study participants reporting HIV positivity from prior testing elsewhere were deemed HIV+ only if written medical documentation of such status was provided. After counseling and consent, participants without such documentation, or of unknown or prior HIV negativity, were HIV tested by standard methods and procedures, including for discordant results, as described previously [13]. Volunteers who declined HIV testing and lacked written confirmation of prior positivity were classified as having HIV-unknown status (HIV^unk^).

### Analysis and statistics

We aimed to describe the prevalence of ASIL and determine associated factors. All statistical tests were 2-sided, and results considered significant when *p* values were ≤0.05. Chi-square for categorical data were used in comparing participants categorized by the gender identities of BM, GM, and TGW. Odds ratios (OR) with 95% confidence intervals (95% CI) and *p* values were calculated for univariate analyses of associations between ASIL and each factors of interest. Multiple logistic regression was performed for variables with univariate *p* values of <0.05, as well as additional variables of interest despite univariate *p* values >0.05. Statistical analysis was by Stata/IC for Windows software, Version 10.0 (StataCorp LP, College Station, TX, USA).

## Results

### Study population, sexual behavior/infection, HIV status

Approximately 400 hundreds clients attended PIMAN center during July 2012 –January 2013. Among half of the clients who declined to participate, the major reasons given were embarrassment to have an anal examination (~40%), concern for pain during the proctoscopic procedure (~25%), not having receptive anal intercourse in the prior 6 months (~20%), and lack of interest or time to participate (~15%).

Of the 200 study participants, 30 (15%) self-identified as BM, 85 (42.5%) as GM, and 85 (42.5%) as TGW (Table 1). The overall mean age was 27.2 years (range 18–54). More than half (59%) were employed and 30% were students. The mean age of sexual debut was 16.8 years. The mean duration of sexually activity was 10.4 years, and median number of sexual partner in the prior 6 months was 3. A strictly anal-receptive sexual role was reported by 121 (60.5%) of participants.

**Table 1:** Selected demographic, sex-behavioral, and other risk factors among men who have sex with men, upon study enrollment, by gender-identity, in Chiang Mai, Thailand 2012–2013.

Characteristics	Gender identity ^a^				
	BM	GM	TGW	Total	*p* value
	N = 30	N = 85	N = 85	N = 200	
	No. (%)	No. (%)	No. (%)	No. (%)	
**Demographic information** ^b^					
**Age (years)**					
<20	4 (13)	8 (9)	15 (20)	**27 (14)**	0.05
≥20 –<30	19 (63)	40 (47)	41 (55)	**100 (53)**	
≥30 –<40	7 (23)	27 (32)	14 (19)	**48 (25)**	
≥40	0	10 (12)	5 (7)	**15 (8)**	
Mean/median	25.2/24	29.5/27	25.6/23	**27.2/25**	
Range	18–36	18–54	18–48	**18–54**	
**Main occupation**					
Unemployed	8 (27)	4 (5)	10 (12)	**22 (11)**	<0.01
Student	5 (17)	23 (27)	32 (38)	**60 (30)**	
Employed	17 (57)	58 (68)	43 (51)	**118 (59)**	
**Sexual behavior**					
**Receptive anal intercourse prior 6 mos.** ^c^	30 (100)	85 (100)	85 (100)	**200 (100)**	
**Anal intercourse prior 6 mos.** ^b^					
Yes	24 (80)	78 (92)	80 (94)	**182 (91)**	0.31
No	3 (10)	4 (5)	4 (5)	**11 (5)**	
Declined to answer	3 (10)	3 (4)	1 (1)	**7 (3)**	
**Sexual role** ^b^					
Insertive only & both insertive/receptive	25 (83)	45 (53)	9 (11)	**79 (40)**	<0.001
Receptive Only	5 (17)	40 (47)	76 (89)	**121 (61)**	
**Age of sexual debut (years)** ^b^					
<18	13 (43)	46 (54)	60 (71)	**119 (60)**	0.05
18–20	13 (43)	28 (33)	17 (20)	**58 (29)**	
≥21	4 (13)	11 (13)	7 (8)	**22 (11)**	
Missing value	0	0	0	**1**	
**Frequency of intercourse prior 6 mos.** ^b^					
≤2 times/week	14 (47)	73 (86)	54 (64)	**141 (71)**	<0.001
≥3 times/week	16 (53)	12 (14)	31 (36)	**59 (30)**	
**Number of sexual partners prior 6 mos.** ^b^					
≤1	6 (22)	23 (28)	21 (25)	**50 (26)**	0.81
≥2	21 (78)	59 (72)	63 (75)	**143 (74)**	
Missing value	3	3	1	**7**	
**Condom use during sex prior 6 mos.** ^b^					
Never/sometimes	11 (41)	21 (26)	36 (44)	**68 (36)**	0.04
Almost every time	16 (59)	60 (74)	45 (56)	**121 (64)**	
Missing value	3	4	4	**11**	
**Rectal douching in prior 6 mos.** ^b^					
Yes	15 (50)	51 (50)	67 (79)	**133 (67)**	<0.01
No	15 (50)	34 (40)	18 (21)	**67 (34)**	
**Other variables**					
**Probable STD symptoms in prior 6 mos.** ^c^					
Yes	22 (73)	60 (71)	64 (75)	**146 (73)**	0.79
No	8 (27)	25 (29)	21 (25)	**54 (27)**	
**Ever smoked in lifetime** ^b^					
Ever	25 (83)	41 (48)	57 (67)	**123 (62)**	<0.01
Never	5 (17)	44 (52)	28 (33)	**77 (39)**	
**Use of estrogen hormone** ^b^					
Ever	6 (20)	22 (26)	71 (84)	**99 (50)**	<0.001
Never	24 (80)	63 (74)	14 (16)	**101 (51)**	
**HIV infection status**					
Positive	4 (13)	30 (35)	15 (18)	**49 (25)**	0.05
Negative	21 (70)	45 (53)	56 (66)	**122 (61)**	
Unknown	5 (17)	10 (12)	14 (16)	**29 (15)**	

^a^ BM = bisexual men, GM = gay men, TGW = transgender women

^b^ Data reported by participant upon enrollment using Computer-Assisted Self Interview (CASI)

^c^ Data reported by participant upon enrollment by face-to-face interview with research physician

Among TGW, exclusive receptive anal intercourse (89%) and rectal douching (79%) was more common than among BM and GM (*p* < 0.001) as well as a use of estrogen hormones (84%; *p <0*.*01*. Table 1). Among GM, condom use almost/every time (74%) was more common than among BM and TGW (*p* = 0.04). Symptoms of probable STDs in the prior 6 months were similar between all three groups. Although the overall rate of ever having smoked was 61.1%, it was more common in the BM group (83%; *p* < 0.01). Physical examination by study physicians found 9 of 200 (4.5%) participants to have genital warts (1 of 30 BM, 6 of 85 GM, and 2 of 85 TGW). Eight of these nine with genital warts were infected with one more hr-HPV types. The proportion HIV+ among subjects with confirmed testing was 13% in BM, 35% in GM, and 18% in TGW.

Overall, ASIL were detected in 86 (43%) of subjects, of which 28 (14.2%) were classified with ASCUS, 1 (0.5%) with ASC-H, 56 (28.4%) with LSIL, and 1 (0.5%) HSIL. The average age of subjects with ASIL was 28.9 years with median of 27.5 years while the average age of subjects with negative cytologic classification was 25.9 years with median of 24 years (*p* = 0.01).

**Fig 1:**
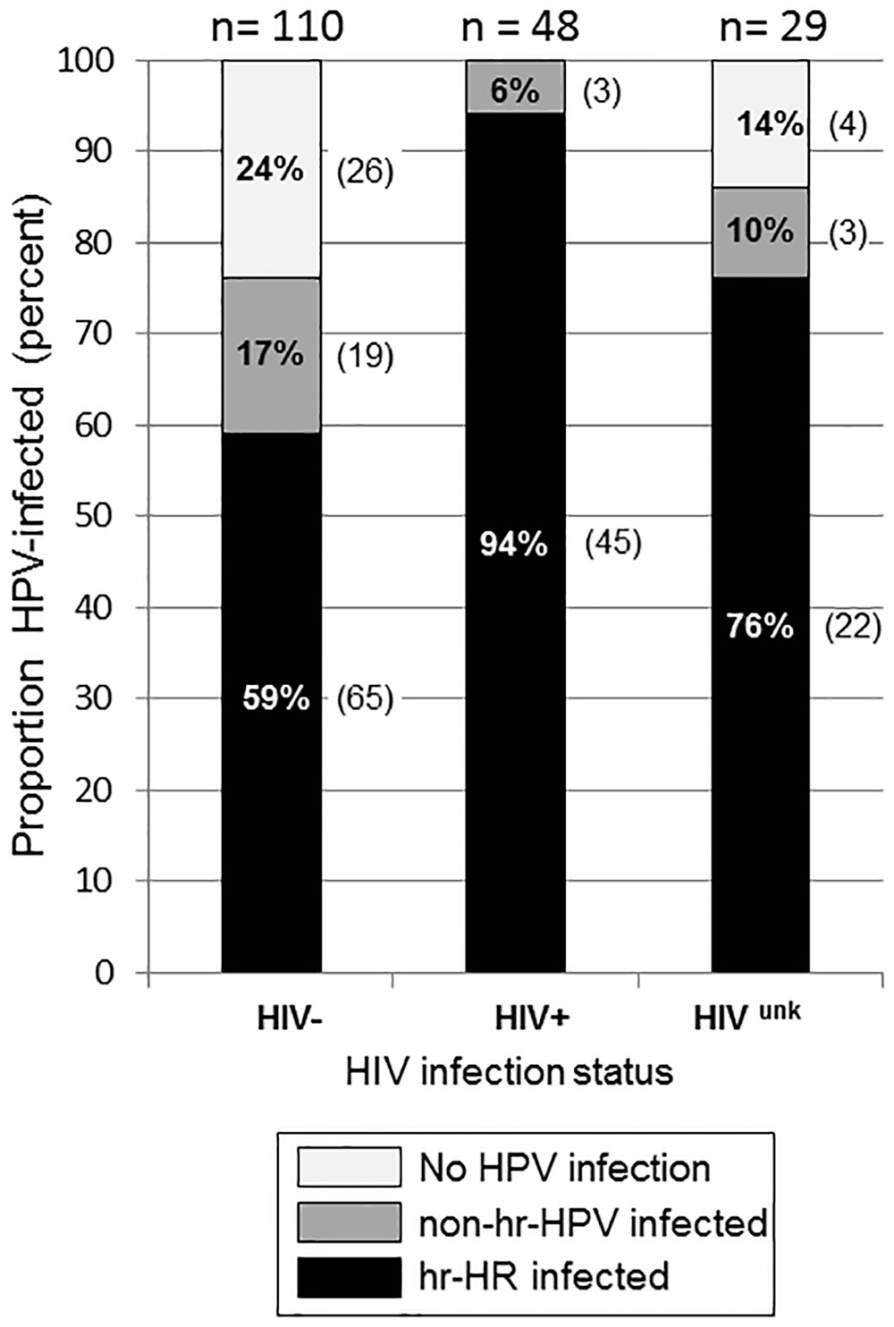
Proportion of subjects without HPV infection, with non-high-risk HPV (non-hr-HPV), and with high risk HPV (hr-HPV), by HIV status of negative (HIV–), positive (HIV+), and unknown (HIV^unk^) among 187 bisexual men, gay men, and transgender women.

**Fig 2:**
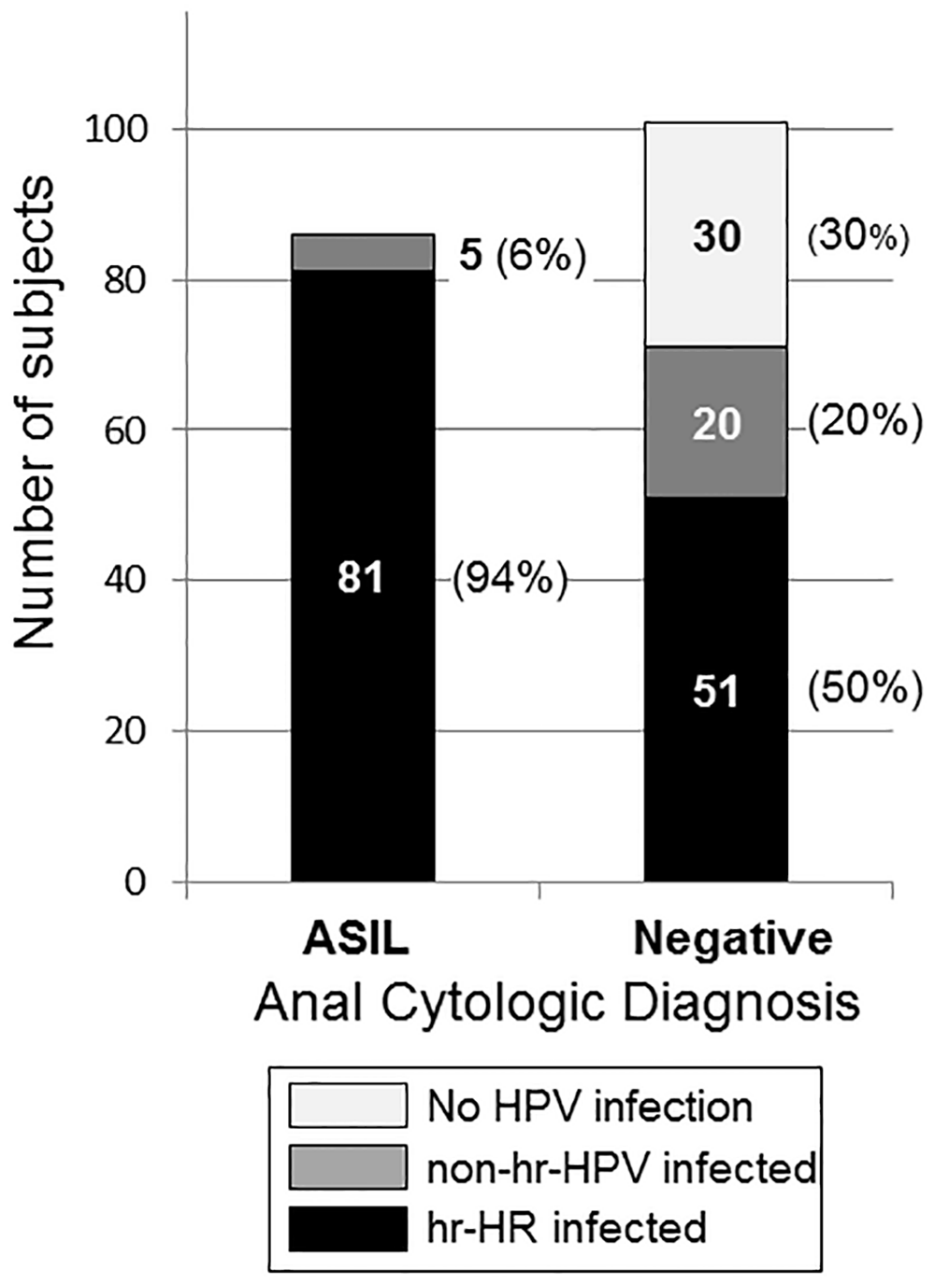
Number of subjects HPV-uninfected, infected with non-high-risk HPV (non-hr-HPV), and infected with high-risk HPV (hr-HPV), among 187 subjects, by anal cytologic diagnosis with anal squamous intraepithelial lesions (ASIL) versus negative.

Three of 200 (1.5%) anal specimens were inadequate for HPV DNA amplification and 10 (5%) were positive for HPV infection by PCR but unable to run HPV typing by Linear Array. HPV infection with identifiable type was detected in 79.7% (157/197) of adequate samples. One or more hr-HPV types were found in 67.0% (132/197). The prevalence of HIV infection was 28.6% (49/171) among those with known HIV status. HIV-testing refusers (HIV^unk^) numbered 29 (14.9%). Of 49 HIV+ participants, 48 anal-swab samples were adequate to determine HPV typing. All HIV+ were infected with one or more HPV types of either high risk (hr-HPV) or non-hr-HPV. Most of them (94%) were infected with hr-HPV. Those of HIV–and HIV^unk^ status had lower proportions of hr-HPV infection (Fig 1). ASIL was found exclusively in participants with HPV infection. A majority (94%; 81/85) of those who had ASIL, found a concurrent hr-HPV infection. Among participant with negative anal cytology, hr-HPV infection was detected in only 46%. (51/111) (Fig 2).

On univariate analysis, older age ≥30 years, gender identity as GM and TGW, rectal douching, anal symptoms in prior 6 months, genital warts, HIV positivity, and hr-HPV infection were associated with ASIL (*p* ≤ 0.05) (Table 2). On multiple logistic regression analysis in which these significant factors on univariate analysis (except for genital warts; see discussion) were included, along with insertive or receptive anal intercourse and condom use (as the factors of interest; see method), only HIV positivity (*p* = 0.01) and hr-HPV infection (*p* = 0.002) were associated with ASIL.

**Table 2:** Univariate Analysis and Multiple Logistic Regression Analysis of Factors Possibly Associated with Anal Squamous Intraepithelial Lesions (ASIL) in 200 Men Who Have Sex with Men in Chiang Mai, Thailand.

Characteristics	Total	ASIL	Univariate analysis		Multiple Logistic Regression Model	
	N = 200	n = 86 (% from N)	OR ^a^ (95% CI)	P-value	AOR ^a^ (95% CI)	P-value
**Demographic information** ^c^						
**Age (yrs.)**						
<20	**27**	8 (30)	Ref			
≥20 –<30	**100**	42 (42)	1.47 (0.59–3.45)	0.41	3.14 (0.92–10.76)	0.07
≥30 –<40	**48**	26 (54)	2.81 (91.03–7.65)	**0.04**	3.42 (0.82–14.18)	0.09
≥40	**15**	10 (67)	4.75 (1.23–18.41)	**0.02**	2.30 (0.37–14.14)	0.37
**Gender identity** ^b^ ^c^						
BM	**30**	6 (20)	Ref			
GM	**85**	44 (52)	4.29 (1.59–11.56)	**0.004**	2.12 (0.45–9.88)	0.34
TGW	**85**	36 (42)	2.94 (1.09–7.93)	**0.03**	2.55 (0.48–13.48)	0.27
**Sexual behavior**						
**Sexual role** ^c^ ^f^						
Insertive only & Both	**79**	37 (47)	Ref			
Receptive Only	**121**	49 (40)	0.77 (0.44–1.37)	0.38	0.42 (0.17–1.27)	0.13
**Age sexual debut (years)** ^c^						
<18	**119**	55 (46)	Ref			
18–20	**58**	23 (40)	0.76 (0.40–1.45)	0.41		
≥ 21	**22**	7 (32)	0.54 (0.21–1.43)	0.22		
**Frequency of sexual intercourse prior 6 mos.** ^c^						
≤2 times/week	**141**	61 (43)	Ref			
≥3 times/week	**59**	25 (42)	0.96 (0.52–1.78)	0.91		
**Number sexual partners prior 6 mos.** ^c^ ^e^ ^f^						
≤1	**50**	16 (32)	Ref			
≥2	**143**	67 (47)	1.87 (0.95–3.69)	0.07	1.26 (0.48–3.32)	0.63
**Condom use prior 6 mos.** ^c^ ^e^ ^f^						
Never/sometimes	**68**	24 (35)	Ref			
Almost every time	**121**	59 (49)	1.74 (0.95–3.22)	0.08	1.40 (0.58–3.34)	0.45
**Rectal douching prior 6 mos.** ^c^						
No	**67**	21 (31)	Ref			
Yes	**133**	65 (49)	2.09 (1.13–3.88)	**0.02**	1.69 (0.67–4.25)	0.26
**Probable STD sign or symptom prior 6 mos.** ^d^						
No	**54**	18 (33)	Ref			
Yes	**146**	68 (47)	1.74 (0.91–3.35)	0.1		
**Anal symptom(s) prior 6 mos.** ^d^						
No	**66**	22 (33)	Ref			
Yes	**134**	64 (48)	1.83 (0.99–3.38)	**0.05**	1.60 (0.66–3.85)	0.3
**Penile symptoms prior 6 mos.** ^d^						
No	**183**	77 (42)	Ref			
Yes	**17**	9 (53)	1.55 (0.57–4.20)	0.39		
**Other variables**						
**Genital warts**						
Absent	**191**	78 (41)	Ref			
Present	**9**	8 (89)	11.38 (1.39–92.87)	**0.02**		
**Ever smoked** ^c^						
Never	**77**	32 (42)	Ref			
Ever	**123**	54 (44)	1.1 (0.62–1.96)	0.75		
**Estrogen hormone use**						
Never	**101**	46 (45)	Ref			
Ever	**99**	40 (40)	0.81 (0.46–1.42)	0.46		
**HIV infection status**						
Negative	**122**	36 (29)	Ref			
Positive	**49**	38 (78)	8.25 (3.80–17.92)	**<0.001**	3.99 (1.36–11.73)	**0.01**
Unknown	**29**	12 (41)	1.69 (0.73–3.89)	0.22	1.49 (0.48–4.55)	0.49
**HPV infection** ^f^						
No HPV infection	**30**	0				
non-hr-HPV infection	**35**	5 (14)	Ref			
hr-HPV infection	**132**	81 (61)	6.35 (2.24–17.99)	**<0.001**	7.06 (2.06–24.18)	**0.002**

^**a**^ OR = odds ratio by univariate analysis; AOR = adjusted odds ratio by multiple logistic regression analysis.

^**b**^ BM = Bisexual men, GM = Gay men, TGW = Transgender women

^**c**^ Data reported by participant upon enrollment using Computer-Assisted Self Interview (CASI)

^**d**^ Data reported by participant upon enrollment by face-to-face interview with research physician

^**e**^ Missing values in this group.

^**f**^ Three anal specimens were inadequate for HPV DNA amplification. Ten specimens were positive for HPV infection by PCR but unable to determine the typing using Linear Array.

^g^ Parameter forced into multiple logistic analysis after non-significant univariate result

## Discussion

Our finding of associations of ASIL in MSM with concurrent hr-HPV infection and with HIV-positivity was not unexpected [8] [9] [18], and of uncertain predictive value for future progression into anal cancer. But as the first report of anal cytology in this population from northern Thailand, what was surprising was an overall rate of ASIL of 43.0%, much higher than the 12.6% reported in Bangkok, Thailand among 123 HIV+ and 123 HIV–patients, using the same cytologic categories as we did (negative versus ASIL, defined as ASCUS or worse) by Phanuphak, *et al*. [19]. This Bangkok research group also reported histologic (biopsy) diagnoses of high-grade anal intraepithelial neoplasia (HGAIN) in 18.9% at their baseline assessment (for which our study could not provide comparison, as we did not perform histologic examinations of anal tissue). Another Bangkok study by Li, *et al*., also lacking histologic diagnosis, reported a prevalence of ASIL of 33.9% among HIV+ MSM subjects [20].

Previous studies elsewhere in Asia also reported lower rates of ASIL among MSM than we found. In Beijing, China, Yang *et al*. found a 37.9% ASIL prevalence in a similar HIV+ population [21], while in Taiwan, Cheng *et al*. reported 28.7% [22]. In a study from India, Arora *et al*. found a 35% ASIL prevalence in the HIV+ and 20% in the HIV–[23]. All of these non-Thai studies lacked histologic confirmation of diagnosis, as we did as well. Our 78% prevalence of ASIL among HIV+ MSM was similar to some series in western countries [9] [24] [25], which, unlike our study, did report both abnormal cytologic findings as well as histologic diagnoses.

Our univariate analyses examined multiple factors of potential association with progression to anal cancer, as reported previously [4] [5] [10]. Some of the variables not found to be significantly associated with ASIL may possibly have resulted from our relatively small sample size and a study population manifesting only the early stages of ASIL. In addition, we found no association of ASIL with participants reporting the use of exogenous estrogen hormone, despite its having been reported as a risk factor for cervical cancer due to its potential effect on the progression of HPV infection [26] [27]

We did find the presence of genital warts to be strongly associated with ASIL on univariate analysis. Despite previous reports that such warts are risk factors for anal cancer, this clinical variable was removed from our multiple logistic regression model to avoid its potential confounding influence. Genital warts are caused by non-carcinogenic hr-HPV types 9 or 11 and in this study all 8 participants who had genital wart with ASIL found to have hr-HPV infection.

In addition to those mentioned above, there were other limitations in this study that may limit generalizability of our findings to other MSM in northern Thailand. First, our high prevalence of ASIL could be explained by a non-representative population, recruited from an HIV voluntary counseling and testing center, at very high risk for HIV and other STDs including HPV. This unrepresentativeness may also have been the result of our selection criteria requiring a history of receptive anal intercourse within the 6 months prior to enrollment. Another reason for an unrepresentative sample of clients might have been the refusal by roughly half of the PIMAN Center clients invited to enroll in the study to do so.

Second, we found inconsistent information regarding receptive anal intercourse in the prior 6 months, with all 200 enrollees reporting it in personal face-to-face interviews, but only 182 reporting any anal intercourse to the CASI. We did not exclude these 18 participants with such discordancy for this study inclusion criteria, aiming to follow the equivalent of an “intention-to-treat” analysis. However, this would have the likely effect, if any, of lowering risk for acquiring HPV, rather than raising it.

Third, there are limitations to anal “Pap-smear” cytology, when evaluated against the accepted gold standard of high-resolution anoscopy (HRA) and biopsy of lesions visualized [15]. Cytology detects ASIL with a sensitivity of only 69–87% and a specificity of 47–82% in HIV+ MSM, and 47–55% and 76–92%, respectively, in HIV–MSM, compared to histology diagnoses by biopsy [28] [25] [29] [30]. This may be due to insufficient, incomplete, or improper swabbing of the anal epithelium, which is more difficult than for cervical swabbing because the anal mucosal folds differ from the smooth mucosa of the uterine cervix. Thus, we may have underdiagnosed or missed some cytologic abnormalities.

Lastly, almost all abnormal cytology found in the study was early stage (ranging from ASCUS to LSIL), with only one participant (0.5%) with HSIL. Previous natural history studies indicate that HPV infections may spontaneously clear to become undetectable by sensitive diagnostic techniques, although this may be less common in HIV+ patients [11] [31]. Lesions in an unknowable number of our participants with early-stage ASIL may regress over time, and not progress to anal cancer. Thus, our finding of a high rate of ASIL may not necessarily predict for a similarly high risk of future anal cancer. However, this rate of ASIL in such young subjects (mean age = 28.9 years) is worrying because of the remaining lifespans ahead during which progression might occur. For example, Chiao, *et al*., reported anal cancer at a mean age of 49 years among HIV+ MSM, and a mean age of 63 for HIV–MSM [32]. Without any anal cancer found in our study, it is not possible to make comparison to these findings.

Clearly, in routine health care, anal cytology is a more convenient and less invasive, if less efficient, routine screening method for anal pathology, compared to HRA with biopsy when indicated. However, anal cytology may be improved by repetition every 12 months among ASIL-negative, HIV+ MSM, or every 2–3 years for MSM who are HIV–, as suggested by Palefsky, *et al*.[28] and Chin-Hong, *et al* [33] [34]. For any found ASIL-positive, they recommend HRA with tissue biopsy, with which we concur.

Our findings of high hr-HPV infection rates indicate that HPV vaccination should be offered to young MSM in Thailand as early as possible, before their high-risk behavior results in infection, after which the vaccine will not protect against type(s) already infecting them. This conforms to the recommendations of the U.S. Centers for Disease Control and Prevention, and its Advisory Committee on Immunization Practices, that HPV vaccine should be offered to young MSM, especially those HIV+ [35]. Moreover, some advocate vaccination even after cytologic abnormalities are detected, because there will still be protection from vaccine antigens not already infecting the patients [36] [37].
